# Association of full blood count findings with risk of mortality in children with *Klebsiella pneumoniae* bloodstream infection at a south african children’s hospital

**DOI:** 10.1186/s12887-023-04104-z

**Published:** 2023-06-17

**Authors:** Johanna T Shapaka, Rudzani Muloiwa, Heloise Buys

**Affiliations:** 1 Department of Paediatrics and Child Health, University of Cape Town, and Red Cross Children’s Hospital, Klipfontein Road, Rondebosch, Cape Town, Western Cape, Cape Town, 7700 South Africa; 2 Division of Ambulatory and Emergency Paediatrics, Red Cross Children’s Hospital, Klipfontein Road, Rondebosch, Cape Town, South Africa

**Keywords:** Klebsiella pneumoniae bloodstream infection, HIV, Children, Full blood counts, Africa

## Abstract

**Background:**

Bloodstream infection (BSI) caused by *Klebsiella pneumoniae* (KP), is a leading cause of hospital-associated childhood mortality. There are limited data on how poor outcomes of KPBSI can be predicted in poorly resourced areas. This study aimed to assess if the profile of differential counts from full blood counts (FBC) taken at two time points in children with KPBSI could be used to predict the risk of death.

**Methods:**

We conducted a retrospective study of a cohort of children admitted to hospital between 2006 and 2011 with KPBSI. FBC collected within 48 h (T1) of blood culture and 5–14 days later (T2), were reviewed. Differential counts were classified as abnormal if they were higher or lower than laboratory ranges for normal results. The risk of death was assessed for each category of differential counts. Risk ratios adjusted (aRR) for potential confounders were used to estimate the effect of cell counts on risk of death using multivariable analysis. Data were stratified by HIV status.

**Results:**

Of 296 children, median age 5 (IQR:2–13) months, 82 were HIV -infected. Ninety-five (32%) children with KPBSI died. Mortality in HIV-infected and uninfected children was 39/82 (48%) and 56/214 (26%), respectively (p < 0.001). Independent associations with mortality were observed with leucopenia, neutropenia and thrombocytopenia. Risk of mortality in HIV-uninfected children with thrombocytopenia at T1 and T2 was aRR 2.5 (95% CI: 1.34–4.64) and 3.18 (95% CI: 1.31–7.73) respectively, whereas the mortality risk in the HIV-infected group with thrombocytopaenia at T1 and T2 was aRR 1.99 (95% CI: 0.94–4.19) and 2.01 (95% CI: 0.65–5.99) respectively. Neutropenia in the HIV-uninfected group at T1 and T2, showed aRR 2.17 (95% CI: 1.22–3.88) and aRR 3.70 (95% CI 1.30-10.51) respectively, while in the HIV-infected group, they were aRR 1.18 (95% CI 0.69–2.03) and aRR 2.05 (95% CI 0.87–4.85) at similar time points. Leucopenia at T2 was associated with mortality in HIV-uninfected and HIV-infected patients, aRR 3.22 (95%CI 1.22–8.51) and aRR 2.34 (95% CI 1.09–5.04) respectively. Persistent high band cell percentage at T2 in HIV-infected children indicated a risk of mortality of aRR 2.91 (95% CI 1.20–7.06).

**Conclusion:**

Abnormal neutrophil counts and thrombocytopenia are independently associated with mortality in children with KPBSI. In resource-limited countries haematological markers have the potential to predict KPBSI mortality.

**Supplementary Information:**

The online version contains supplementary material available at 10.1186/s12887-023-04104-z.

## Background

Gram-negative septicaemia caused by *Klebsiella pneumoniae* bloodstream infections (KPBSI), is a leading cause of childhood mortality globally [1, 2] *Klebsiella pneumoniae* (KP*)* infection is the second most prominent Gram-negative infection in hospital- and intensive care unit (ICU)-based infections. Between 2010 and 2015, KP was among the top five causes of nosocomial infections among 703 ICUs in 50 countries evaluated by the International Nosocomial Infection Control Consortium (INICC) in 2016 [3]. There is however a paucity of data on KPBSI in sub-Saharan Africa, thus the true burden, may be underestimated.

In 2013, the World Health Organization (WHO) reported that overall, sepsis accounted for almost 6.3 million deaths worldwide in children under 5-years of age. The majority of deaths were in low-middle-income countries (LMIC) [2, 4]. The prevalence of sepsis in 2013 was estimated at 6.2% in Europe and 23.1% in Africa [2, 4]. Emerging data describe a strong association of acquiring KPBSI with hospitalization, compounded by risk factors such as malnutrition and HIV infection which are highly prevalent in children in low- and middle-income (LMIC) settings [5].

KPBSI is diagnosed by the isolation of KP in blood culture. Multiplex real-time PCR -based testing methods also exist for detecting bacterial pathogens in blood as an alternate option but are less cost-effective and cannot replace conventional blood culture testing [6]. Though KPBSI poses a high risk for death, there are currently no easily accessible tools for predicting this risk for children in (LMICs). KPBSI may induce an overall leucocytosis, with a predominant left-sided shift [7].

Considering that full blood counts (FBC) are readily accessible in LMIC, this study aimed to describe various cell lines contained in a FBC analysis of children younger than 13 years of age with confirmed KPBSI seen at a children’s hospital. Given the high burden of HIV in Sub-Saharan Africa and the overall impact of HIV-infection on mortality, the findings were stratified by HIV status.

## Methods

### Study setting

The study retrospectively analysed data from a previously published study that investigated the outcome of children with KPBSI [5]. The study was conducted at the Red Cross Children’s Hospital (RCCH) in Cape Town, South Africa. RCH is a 282-bedded public children’s hospital, serving all children up to 13 years of age. RCCH delivers the full range of tertiary level paediatric care, but no dedicated newborn care. Newborn infants are referred in if they require cardiac or other specialised surgery.

The study included patients that were admitted at RCCH between 2011 and 2016 who had laboratory confirmed KP bloodstream infection. At the time of sampling the blood culture, the patient was initiated on empiric antibiotics to treat suspected systemic sepsis; during the study period, these antibiotic recommendations were taken from the study site’s antimicrobial guidelines. Hence briefly, for a community-acquired episode of systemic sepsis, a combination of ampicillin and gentamicin were used; for children with presumed healthcare-associated sepsis, a combination of piptazobactam and amikacin would have been used. The choice of antibiotic was reviewed when laboratory sensitivities became available, and in the case of ESBL KP, a carbapenem would have been used. Patients were included in the study if they had FBC samples taken within two days of the sample that cultured positive for KP. In addition, the children needed to have laboratory confirmed HIV status.

### Data collection

Methods for data collection have been previously published [5]. Briefly, the collated database recorded the patients’ demographic data, clinical history, HIV status, nutritional status and laboratory results. Specifically, the results included FBC white cell counts (WCC), absolute neutrophil counts (ANC), band cell counts, band cell percentage and platelet (PLT) counts. The FBC findings were collected at two time points. The first sample needed to have been collected within 48 h of the blood sample that grew KP (T1). The second FBC was taken between 5 and 14 days of the sampling of the blood sample (T2) that cultured KP. T2 was chosen as a time period that was deemed to reflect sufficient time for antibiotics to have meaningful effect.

A child younger than 18-months-of-age who tested positive for HIV on two separate HIV PCR tests was considered HIV-infected or HIV-positive [8]. A child older than 18 months who tested positive on two separate specimens on an HIV ELISA test was considered HIV-infected [8]. A KPBSI was regarded as hospital-acquired if it occurred at least 48 h after hospital admission or within one week of discharge with evidence of infection related to previous infection [9]. World Health Organization (WHO) weight-for-age Z-scores (WAZ) were used to assess nutritional status. A WAZ between − 2 and − 3 standard deviations (SD) was classified as moderate underweight-for-age (UWFA), and WAZ less than − 3 SD was classified as severe UWFA [10].

### Data analysis

The outcome of interest was dying during the current admission. Cut-off values to interpret FBC findings in the study were guided by laboratory values reported in the literature. Overall leucocytosis was defined as a WCC ≥ 15 × 10^9^ cells/L, and leukopenia ≤ 5 × 10^9^ cells/L. Neutrophilia was defined as ANC ≥ 10 × 10^9^ cells/L, and neutropenia ≤ 1.5 × 10^9^ cells/L. A band cell percentage was considered normal when ≤ 10% and high when ≥ 10%. Absolute band counts were considered abnormally high if > 0.250 × 10^9^ cells/ L and within normal if < 0.250 × 10^9^cells. Normal platelet counts range between 150 and 449 × 10^9^ cells/L, with a count ≤ 150 × 10^9^ cells/L considered thrombocytopenia and a count ≥ 500 × 10^9^ cells/L considered thrombocytosis [11–15].

Frequencies of categorical variables including age, sex, nutritional status, ESBL-type were described as percentages. Continuous data were expressed as medians and interquartile ranges (IQR). The Wilcoxon signed-rank test was performed to compare changes between T1 and T2 of the distribution of cell counts. Categorical descriptors were made for the individual FBC components in three primary categories: normal, high and low, as defined by laboratory cut-off values. The *χ*2 test or Fisher’s exact test were used to assess the strength of association between two categorical variables as appropriate.

Factors showing some association with poor outcome on a univariate analysis were included in a generalised Poisson regression model used to estimate independent association between different cell counts and mortality. Effects from this confounder-adjusted multivariable model were presented as adjusted risk ratios (aRR) with 95% confidence intervals (CI). The final model adjusted for age-in-months, presence of extended-spectrum β- lactamase-producing *Klebsiella pneumoniae* and sex. As there was a difference between the timing of FBC sampling between survivors and those who died, the day of sampling since the day of blood culture was also included in the model for adjustment. All analyses were conducted stratified by HIV status and results presented in text, frequency tables and graphically. A significance level was at a two-sided  < 0.05 for all analyses. Data were analysed using STATA version 16 (StataCorp LP).

### Ethical considerations

The Health Sciences Human Research Ethics Committee of the Faculty of Health Sciences of the University of Cape Town and the Red Cross Children’s Hospital Administration approved this study (HREC Ref: 786/2017). Due to the retrospective nature of the study, the Health Sciences Human Research Ethics Committee of the Faculty of Health Sciences of the University of Cape Town waived the need for written informed consent. The study was conducted in accordance with the Declaration of Helsinki of 2013.

## Results

### Baseline characteristics

**Fig. 1:**
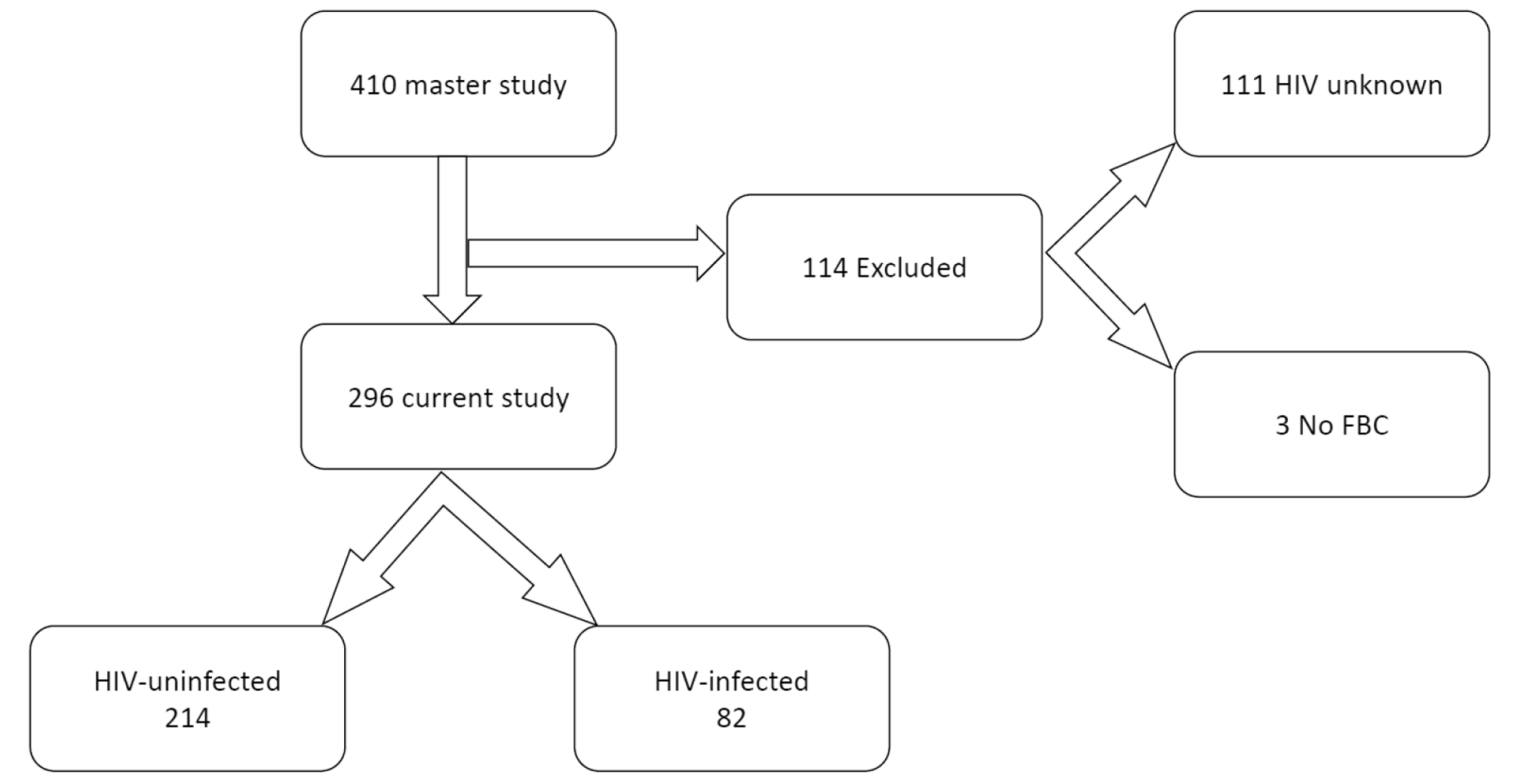
Enrolment flow chart of study participants

Of 410 potential study participants, 296 had sufficient data and were included in the final analysis. Figure 1.

There were 151 (51%) boys in the group while 82 (28%) children were confirmed HIV-infected. Of the whole group, 37 (12.5%) were neonates. In 257 (87%) of the children the blood stream infection was due to an extended-spectrum β-lactamase-producing-*Klebsiella pneumoniae* (ESBL KP). Other baseline characteristics of the study children are summarised in Table 1. Moderate-to-severe underweight-for-age was present in 166 (57%) of the children with 54 (66%) in the HIV-infected group compared to 112 (52) in the HIV-uninfected group; p = 0.036.

**Table 1:** Baseline characteristics of study population (N = 296)

Variable	n (%)
Sex	
Male	151 (51)
Female	145 (49)
Median age in months (IQR)	5 (1–13)
HIV status	
HIV-infected	82 (28)
HIV-uninfected	214 (72)
Nutritional status	
Normal	106 (36)
Moderate-to-severe underweight-for-age^#^	190 (64)
*Klebsiella pneumoniae* strain	
Non-ESBL	39 (13)
ESBL	257 (87)
Type of infection	
Community-acquired	21 (7)
Hospital-acquired	275 (93)
Chronic underlying condition	
None	89 (30.1)
HIV disease	82 (27.7)
Gastrointestinal	42 (14.2)
Cardiac	30 (10.1)
Neoplasm	19 (6.4)
Renal	13 (4.4)
Neurological	8 (2.7)
Tuberculosis	6 (2.0)
Aplastic anaemia	3 (1.0)
Primary immunodeficiency	2 (0.7)
Other	2 (0.6)
Focus of infection	
Nil identified	157 (53)
Pneumonia	93 (32)
Indwelling venous catheter	22 (7.4)
Necrotising enterocolitis	14 (5.8)
Peritonitis	14 (4.8)
Urinary tract infection	12 (4)
Soft tissue	11 (3.7)
Other	19 (6)

ESBL = extended-spectrum β- lactamase-producing *Klebsiella pneumoniae*; Where the percentages do not add up to 100%, patients had more than one focus of infection; IQR- interquartile range; ^#^ weight-for-age-Z-scores < -2 standard deviations

T1 FBC results were available for all 296 participants but only for 178 (60%) at T2. The first FBC sample was taken at median day 0 (IQR 0–0) for both survivors and those who died, p = 0.9008; while the second sample was taken at day 7 (IQR 6–8) in survivors and at 7 (IQR 5.5-7) days in those who died, p = 0.039. The overall mortality in the study population was 95 (32%) out of 296. The mortality was 39 (48%) out of 82 in the HIV-infected group compared to 56 (26%) out of 214 in the HIV-uninfected group, p < 0.001.

### Trends of FBC cell counts at T1 and T2 stratified by HIV status

#### HIV-negative group

**Fig. 2:**
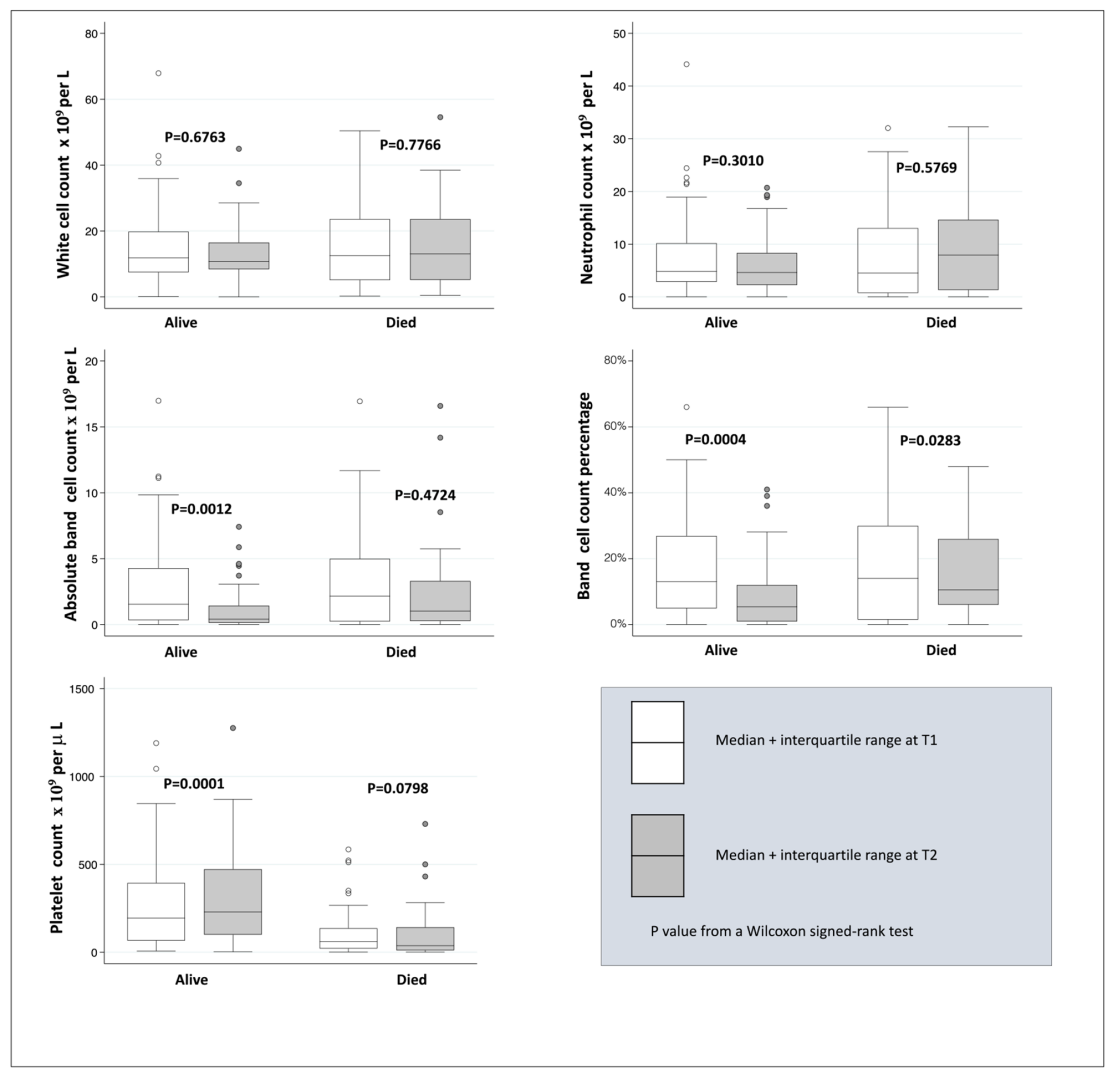
Distribution of blood cell counts in HIV-negative children at T1 (< 48 h) and T2 (5–14 days) after blood culture by survival status

Among HIV-negative patients, platelet counts were low in survivors with median of 66 (IQR 66–394) x 10^9^ cells/L and 60 (IQR 21–136) x 10^9^ cells/L in patients who died. In those who did not die, there was significant change to normal at T2 to a median of 229 (IQR 100–472) x 10^9^ cells/L (p = 0.0001); whereas platelet counts did not improve in children who died with T2 platelet count median of 37 (IQR 11–142) x 10^9^ cells/L, p = 0.0798. Similarly absolute band cell counts improved to normal for surviving children from a median of 1.55 (IQR 0.34–4.28) x 10^9^ cells/L to 0.41 (IQR 0.13–1.43) x 10^9^ cells/L, p = 0.0012. Among the children who died, although there was an improvement from a median of 2.17 (IQR 0.24–4.99) x 10^9^ cells/L at T1 to a median of 1.03 (IQR 0.27–3.3) x 10^9^ cells/L at T2, this change was not significant, p = 0.4724. In the HIV-uninfected children, there was a significant improvement in the median band cell count percentage in both the survivors, from a median band percentage of 13.04% (IQR 4.91–26.9) at T1 to 5.01% (IQR 0.98-12.0) at T2; and in those who died from a median band percentage of 14.04% (IQR 1.50–30.0) at T1 to 10.5% (IQR 6.01-26.0) at T2, p = 0.0004 and p = 0.0283 respectively. No significant change was noted for total white cell counts or neutrophils between T1 and T2 for both HIV-negative survivors and those who succumbed to KPBSI Fig. 2.

### HIV-positive group

**Fig. 3:**
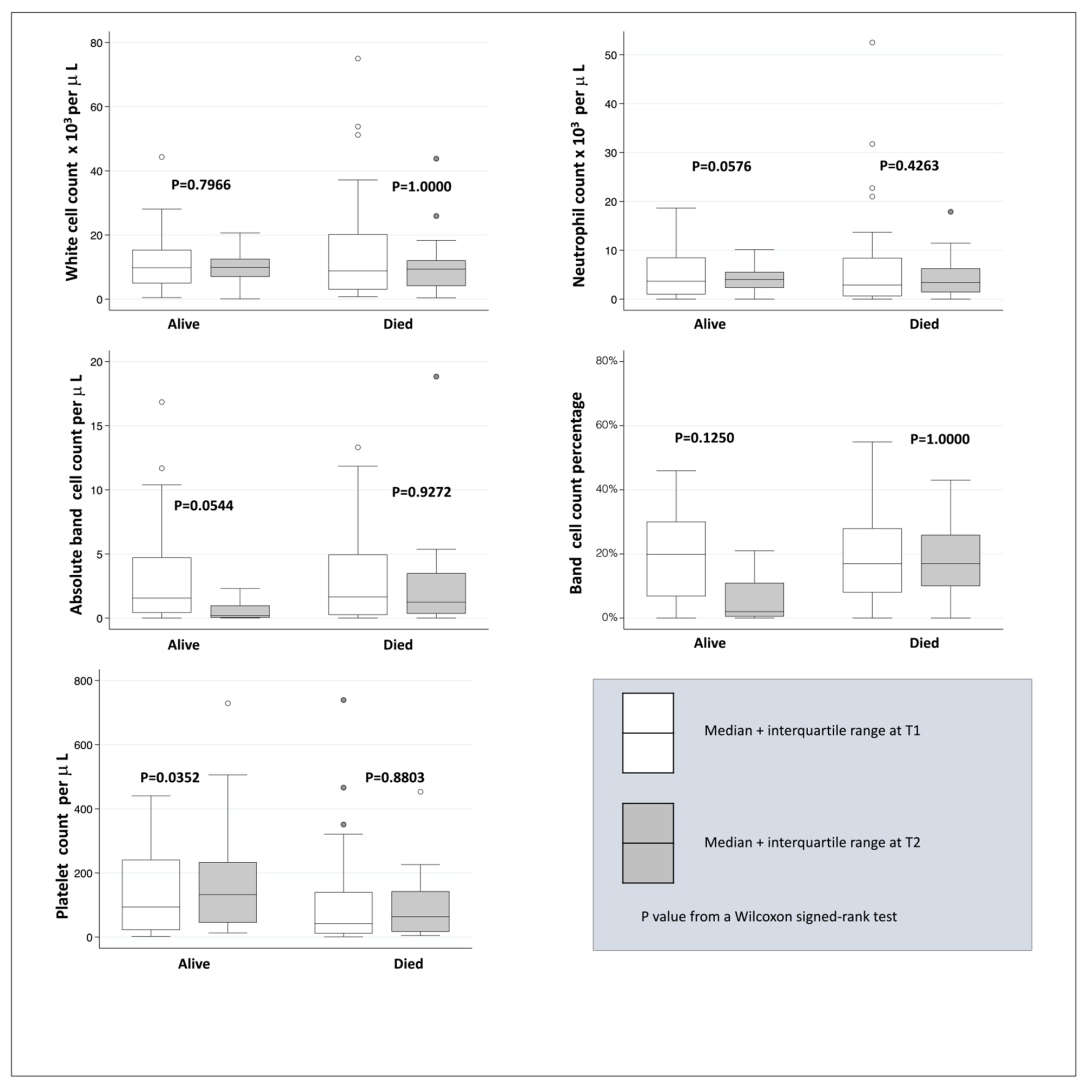
Distribution of blood cell counts in HIV-positive children at T1 (< 48 h) and T2 (5–14 days) after blood culture by survival status

A similar pattern was observed in the HIV-positive group in whom absolute band counts changed from median 1.56 (IQR 0.41–4.73) x 10^9^ cells/L at T1 to 0.19 (IQR 0.35–0.99) x 10^9^ cells/L at T2 (p = 0.0544); while platelet counts changed from median 94 (IQR 22–241) to 134 (IQR 45-233.5) x 10^9^ cells/L in survivors (p = 0.0352). In children who died, band counts remained the same at T1 and T2 with medians of 1.65 (IQR 0.25–4.96) x 10^9^ cells/L and 1.25 (IQR 0.35–3.5) x 10^9^ cells/L respectively, p = 0. 9272. Platelet counts were at a median of 42 (IQR 11–140) and 63.5 (IQR 17–143) x 10^9^ cells/L at T1 and T2 respectively, p = 0.8803. Similarly, WCC and neutrophils did not change for both outcomes at T1 and T2. Figure 3.

### Association between categories of FBC profile and mortality by HIV status

At T2 mortality was independently associated with leucopenia in HIV-uninfected patients, aRR 3.22 (95%CI 1.22–8.51), and in the HIV-infected group, aRR 2.34 (95% CI 1.09–5.04). Independent association with mortality was also seen at T1 for neutropenia in the HIV-uninfected group, aRR 2.17 (95% CI: 1.22–3.88), but a similar pattern was not seen in the HIV-infected group. At T2 neutrophilia seemed to be associated with dying during admission for both HIV-uninfected and HIV-infected group, aRR 4.04 (95% CI 1.65–9.87) and aRR 3.54 (95% CI 1.15–10.93), respectively. A similar independent finding was seen during T2 for neutropenia in the group that were HIV-uninfected, aRR 3.70 (95% CI 1.30-10.51), but not in the HIV-infected group, aRR 2.05 (95% CI 0.87–4.85).

Table 2 shows the other unadjusted and adjusted risk effects of different FBC cell lines on mortality early (T1) and later (T2) in the course of KPBSI in both HIV-infected and -infected children.

**Table 2:** Adjusted and unadjusted risk ratios for mortality with early (T1) and late (T2) differential count in patients stratified by HIV status

			HIV-uninfected patients						HIV-infected patients				
Period	Risk factor		Risk n/N (%)		RR (95% CI)		aRR (95%CI) #		Risk n/N (%)		RR (95% CI)		aRR (95%CI) #
**T1**	Normal band counts		12/38 (32)		1		1		8/14 (57)		1		1
	Absolute bands high		36/137 (26)		0.83 (0.48–1.44)		0.87 (0.50–1.50)		26/51 (51)		0.89 (0.52–1.52)		0.75 (0.45–1.24)
	Normal band percentage		20/68 (29)		1		1		14/24 (58)		1		1
	Band percentage high		28/107 (26)		0.89 (0.55–1.45)		0.92 (0.56–1.50)		20/41 (49)		0.84 (0.53–1.33)		0.77 (0.48–1.24)
	Normal white cell count		19/88 (22)		1		1		14/35 (40)		1		1
	Leucocytosis		23/84 (27)		1.27 (0.75–2.16)		1.26 (0.74–2.14)		14/25 (56)		1.40 (0.82–2.40)		1.50 (0.86–2.61)
	Leucopenia		14/42 (33)		1.54 (0.86–2.77)		1.55 (0.73–2.14)		11/22)		1.25 (0.70–2.25)		1.36 (0.74–2.47)
	Normal neutrophil count		19/94 (20)		1		1		15/30 (50)		1		1
	Neutrophilia		15/48 (25)		1.55(0.86–2.77)		1.60 (0.90–2.84)		7/13 (54)		1.08 (0.58–2.01)		1.19 (0.63–2.24)
	Neutropenia		14/33(33)		**2.10 (1.19–3.70)**		**2.17 (1.22–3.88)**		12/22 (55)		1.09 (0.64–1.85)		1.18 (0.69–2.03)
	Normal platelet count		10/66 (15)		1		1		6/23 (26)		1		1
	Thrombocytosis		3/33(9)		0.6 (0.18–2.03)		0.62 (0.18–2.12)		2/2 (100)		**3.83 (2.44–12.63)**		**5.56 (2.44–12.63)**
	Thrombocytopenia		42/111 (38)		**2.50 (1.34–4.64)**		**2.50 (1.34–4.64)**		29/55 (53)		2.02 (0.97–4.22)		1.99 (0.94–4.19)
			**HIV-uninfected patients**						**HIV-infected patients**				
**T2**													
	Normal band counts		6/33 (18)		1		1		3/14 (21)		1		1
	Absolute bands high		20/61 (33)		1.80 (0.80–4.06)		1.82 (0.85–3.89)		11/20 (55)		2.57 (0.86–7.67)		2.13 (0.75–6.07)
	Normal band percentage		13/60 (22)		1		1		20/68 (29)		1		1
	Band percentage high		13/33 (39)		1.82 (0.96–3.36)		1.64 (0.83–3.24)		13/33 (39)		**3.17 (1.22–8.23)**		**2.91 (1.20–7.06)**
	Normal white cell count		9/66 (14)		1		1		8/26 (31)		1		1
	Leucocytosis		14/44 (32)		**2.28 (1.08–4.83)**		**2.16 (1.05–4.44)**		4/9 (44)		1.44 (0.56–3.70)		1.51 (0.58–3.93)
	Leucopenia		7/21 (33)		**2.44 (1.03–5.78)**		**3.22 (1.22–8.51)**		6/11 (55)		1.77 (0.80–3.94)		**2.34 (1.09–5.04)**
	Normal neutrophil count		7/50 (14)		1		1		8/25 (32)		1		1
	Neutrophilia		12/25 (48)		**3.43 (1.54–7.66)**		**4.04 (1.65–9.87)**		2/3 (67)		2.08 (0.77–5.65)		**3.54 (1.15–10.93)**
	Neutropenia		7/19 (28)		**2.63 (1.06–6.53)**		**3.70 (1.30-10.51)**		4/6 (67)		2.08 (0.92–4.71)		2.05 (0.87–4.85)
	Normal platelet count		6/41 (12)		1		1		3/14 (21)		1		1
	Thrombocytosis		2/28(7)		0.59 (0.12–2.83)		0.68 (0.14–3.21)		1/3 (33)		1.56 (0.23–10.50)		2.27 (0.49–10.42)
	Thrombocytopenia		23/60 (38)		**3.14 (1.30–7.72)**		**3.18 (1.31–7.73)**		14/29 (48)		2.25 (0.76–6.66)		2.01 (0.67–5.99)

T1 = sample taken within 48 h of blood culture; T2 = sample taken 5–14 days after blood culture; RR (95% CI) = relative risk (95% confidence interval); aRR = adjusted relative risk; # Multivariable model adjusted for age-in-months, sex, extended-spectrum beta- lactamase-producing *Klebsiella pneumoniae*, day since blood culture. Statistically significant results are in **bold print**

## Discussion

Our study has indicated that children with KPBSI whose overall band cells and platelets did not show significant improvement between the first 48 h of sampling of a positive blood culture and a period at least five days after had a higher risk of dying irrespective of their HIV status. In addition, this study indicates that persistent neutropenia and thrombocytopenia are independently associated with death in this group of children. HIV-uninfected children had a two and a half to three times risk of dying if they still demonstrated neutropenia and low platelet counts five days from the date of the sampling of the positive blood culture and initiating of antimicrobial therapy. We believe this to be the first time that these findings have been reported and we have not found other published studies to compare. High band cell count percentages often indicate that a severe infection is present. The reduction of these counts over time with clinical improvement in patients suggest that the infection has been brought under control. HIV-infected children, in addition to having HIV viral toxicity, impaired myelopoiesis with maturational deficiencies have severe immune dysfunction and poor antibody response against severe bloodstream infection [22].

KPBSI has been reported to cause a leucocytosis, with a predominant left-sided shift [7, 12]. In a Korean study involving 3 862 infants, 45 cases of early-onset sepsis were described. *Group B Streptococcus* caused 22% (n = 10) of cases and *Escherichia coli* caused 20% (n = 9 cases). The overall mortality was 38% (17/45). Odds ratios adjusted (aOR) for confounders were used to show that Gram-negative sepsis, neutropenia and inactivity were associated with fatality, aOR 42 (95% CI 1.4-1281.8), aOR 46 (95% CI 1.3-1628.7) and aOR 34 (95% CI 1.8-633.4) respectively [16]. The mechanisms of how KP infection affects the platelets, band cells and white cells are not fully understood. Specifically, data on how KPBSI affects the haematological cell lines in children are limited, however the effect of sepsis causing disseminated intravascular coagulation (DIC) has been well described [17]. However, it is postulated that multifactorial components including the depletion of antibodies, the enhanced coagulation, the increased proinflammatory cytokine levels could explain the some of the abnormalities seen in the FBC with KP bloodstream infection. There is some data from a study from Turkey where 62 of 5 535 hospitalised children, with median age 4 years, who were diagnosed with acute DIC were studied. Infection was the cause of the DIC in 59 (95%) children, and severe injury the cause in the other 3 (5%). The infecting pathogens were identified as *Neisseria meningitidis* in four children, Gram-negative pathogens in ten and Gram-positive pathogens in six children. The haematological findings described by this study included FBC counts, prothrombin time (PT), activated partial thromboplastin time APTT), International normalised ratio (INR), fibrinogen and D-dimers. None of the laboratory findings played a predictive role for mortality; specifically, there was no difference in the platelet counts or white cell counts in survivors compared to those who died, p value 0.629 and 0.046 respectively. Of these 62 children, 35 (56%) died including 8 of the 10 children with Gram-negative infection. Multiorgan dysfunction syndrome and acute cardiorespiratory dysfunction were the factors significantly associated with mortality in their multivariate analysis [18]. In contrast, our study demonstrated that if thrombocytopenia among other markers does not recover after therapeutic interventions, particularly if platelet counts remain persistently low at day 5–14 of therapy, the risk of mortality is significantly higher.

The overall mortality in the study population was 95 (32%) out of 296. Persistence of abnormal white cell counts (leucopenia and neutropenia) and thrombocytopenia in the HIV-uninfected patients and the persistence of leucopenia and high band cell counts in the HIV-infected patients were associated with increased mortality. As reported previously, our data demonstrate that HIV is a risk factor for KPBSI mortality [5]. This may be compounded by malnutrition that tends to complicate HIV-infection. In HIV-infection the immunosuppression is due to a significant decrease in CD4 + and CD8 + T-lymphocytes [19]. Furthermore, during the time our data were collected, most of the HIV-infected patients were also malnourished. This dual burden of HIV infection and malnutrition further increase the risk for acquiring Gram-negative infections and the risk of mortality [20]. Furthermore, overwhelming infection with consumption of clotting factors and platelets, and marrow suppression may be at play further setting up a vicious cycle of perpetuating sepsis. This may further explain why neutrophils and platelets remain low and bands remain high, even five-to-fourteen days post initiation of therapy, in KPBSI.

Once the data were stratified by HIV infection, nutritional status was not added to the adjusted model to avoid colinear introduction of confounding as it was strongly associated with HIV status. It has been reported that both HIV infection and Gram-negative sepsis can affect the FBC and are associated with immune dysregulation. Mechanisms in HIV infection are likely multifactorial given the high prevalence of malnutrition and include an effect on haematopoietic production by infection of progenitor cells, infiltration of bone marrow, nutritional deficiencies, autoimmune destruction, other intercurrent infections and drugs which may impair synthesis. On the other hand, thrombocytopenia may be a marker of disease severity as well as a portender of significant host defence impairment. Sepsis may cause increased destruction as well as impair production; in experimental studies in mice, it has been eloquently demonstrated that as thrombocytopenia increases, there is associated increased pro-inflammatory cytokine release, haemorrhage at the primary site of sepsis as well as increased mortality. It is also postulated that thrombocytopaenia is associated with enhanced bacterial dissemination to the sepsis area through inhibition of neutrophil degranulation. Whilst this type of experimental work would be difficult to replicate in human studies, these data eloquently offer mechanisms for the consequences of thrombocytopaenia and neutropenia. Paediatric studies on these mechanisms are sparse [20–23].

This study is limited by its retrospective design which in part explains the 114 potential participants that had to be excluded and the unavailability of T2 FBC data for some of the included children, creating a relatively small final sample size. Certain of the laboratory investigations e.g., C-Reactive protein, procalcitonin, other FBC indices (haemoglobin and mean cell volume) and clotting profiles that could have elucidated some of the causes implicated in mortality e.g., severe bacterial infection and coagulopathy, could not be included in the data analysis as they were not available in many cases and when available, were not available within the stipulated T1 and T2 time points. Furthermore, haemoglobin levels are compounded by age, chronic illness, malnutrition, and therapeutic blood transfusions and would have been difficult to interpret. As can be seen with the stratified analysis, the main underlying disease under investigation was HIV, however some patients had other underlying conditions which may have affected their haematological responses. Moreover, the patients infected with HIV were not all on antiretroviral therapy (ART), as the study was conducted during the era when ART was not readily available to all children. This study is therefore unable to assess the impact of antiretroviral therapy on FBC in the current cohort of children. Children who are virologically suppressed may respond differently to infection from those who are not virologically suppressed [24]. We did not interrogate the effect of antibiotics such as cotrimoxazole and other drugs which may affect the FBC, nor the differences in FBC for patients with documented persistent bacteraemia versus those with documented clearance on blood culture.

One further limitation to note is that neonates do tend to behave differently and are treated differently in neonatal units, and thus have their own cut-off values for the full blood count. In our study, the neonates constituted a very small proportion of the whole group, and the numbers did not allow for a meaningful subgroup analysis. However, from a pragmatic point of view in low-resourced settings, the guidelines we have for infants < 2-months of age do not discriminate between neonates and other very young infants.

We believe that the present study is novel in its approach and findings as we could not find other studies to compare to nor build a robust literature review from published findings, though it is likely that KP infection mechanism on platelets and other cell lines, behaves the same as other Gram-negative organisms. Though there are studies looking at KPBSI, we could not find research data focusing on how KPBSI influences the FBC in children.

Our results potentially offer a means of monitoring patients at risk for deterioration and death, which would involve less strain on constrained resources. Our study was designed from a KPBSI database from a demography which is representative and comparable to many sub-Saharan African countries. This study will hopefully encourage further research that could potentially look prospectively at how Gram-negative organisms such as KP influence the various cell lines, including their impact on HIV-infected children on ART as well as in children who are HIV-exposed. Moreover, having this knowledge on how KPBSI effects the various cell lines, future research could explore how initiation and prompt escalation of therapy could change mortality. The results of this study could therefore be potentially of use in resource-limited countries where there may be poor access to blood cultures or prolonged blood culture result turn-over but may also be deemed useful in higher-income settings.

## Conclusions

Overall, our study suggests that FBC profile, specifically band cell and platelets counts taken at two time periods may be of value in predicting children who are at risk of dying from KPBSI. In resource-limited countries where blood cultures cannot be done readily, or turn-over time is prolonged, readily available haematological markers could be used as predictors of KPBSI associated risk of mortality even in areas with a high burden of HIV infection. Clinicians should be alerted to critically reviewing therapeutic options in children with KPBSI whose full blood count differentials remain persistently abnormal at 5–14 days after starting appropriate antibiotic treatment.

## Electronic supplementary material

Below is the link to the electronic supplementary material.

Supplementary Material 1

Supplementary Material 2

## Data Availability

All data generated or analysed during this study are included in this published article.

## Abbreviations

AIDS: Acquired immuno-deficiency syndrome
ANC: Absolute neutrophil count
95% CI 95%: Confidence interval
CRP: C-reactive protein
DIC: Disseminated intravascular coagulation
DNA PCR: Deoxyribonucleic acid polymerase chain reaction
ESBL: KP extended-spectrum β- lactamase-producing *Klebsiella pneumoniae*
FBC: Full blood count
FE: Fisher’s Exact
ART: Antiretroviral therapy
Hb: Haemoglobin
HIV: Human immunodeficiency virus
ICU: Intensive care unit
INR: International normalized ratio
IQR: Interquartile range
KP: *Klebsiella pneumoniae*. KPBSI *Klebsiella pneumoniae* bloodstream infection
LMIC: Low-middle-income countries.
NHLS: South African National Health Laboratory Services.
PLT: Platelets.
PTT: Partial thromboplastin time.
RCH: Red Cross Children’s Hospital
RNA: PCR ribonucleic acid polymerase chain reaction.
T1: Time 1.
T2: Time 2.
UWFA: Underweight-for-age.
WCC: White cell count.
WHO: World Health Organization.
