# Supplementary material for: Association of full blood count findings with risk of mortality in children with Klebsiella pneumoniae bloodstream infection at a south african children’s hospital
Source: BMC Pediatr. 2023 Jun 17;23:302. doi: 10.1186/s12887-023-04104-z (PMC10276429; doi:10.1186/s12887-023-04104-z)
Supplement: Supplementary file 1 — Supplementary Material 1 [file 12887_2023_4104_MOESM1_ESM.pdf]

```
1 .
2 . * Descriptions

3 . bysort hiv2: tabstat new1wcc new2wcc if hiv2s!=., by(outcome) stats (p50 p25 p75)
```

-> hiv2s = 0

Summary statistics: p50, p25, p75
by categories of: outcome (Outcome)

outcome	new1wcc	new2wcc
Alive	11.85	10.78
	7.5	8.41
	19.8	16.48
Died	12.535	13.08
	5.105	5.2
	23.61	23.6
Total	12.25	11.09
	6.9	7.7
	20.6	18.1

-> hiv2s = 1

Summary statistics: p50, p25, p75
by categories of: outcome (Outcome)

outcome	new1wcc	new2wcc
Alive	9.81	9.9
	4.95	7
	15.39	12.55
Died	8.82	9.35
	3	4.1
	20.26	12.08
Total	8.975	9.9
	4	5.7
	18	12.4

```
4 . bysort hiv2: tabstat new1neut new2neut if hiv2s!=., by(outcome) stats (p50 p25 p75)
```

-> hiv2s = 0

Summary statistics: p50, p25, p75
by categories of: outcome (Outcome)

outcome	new1neut	new2neut
Alive	4.86	4.63
	2.86	2.27
	10.18	8.33
Died	4.525	7.955
	.745	1.33
	13.05	14.62
Total	4.77	4.66
	2.22	2.1
	11.03	10.06

-> hiv2s = 1

Summary statistics: p50, p25, p75
by categories of: outcome (Outcome)

outcome	new1neut	new2neut
Alive	3.69	3.995
	.99	2.31
	8.51	5.56
Died	2.9	3.41
	.62	1.41
	8.45	6.29

Total	3.53	3.735
	.98	1.72
	8.45	6.02

```
5 . bysort hiv2: tabstat new1band new2band if hiv2s!=., by(outcome) stats (p50 p25 p75)
```

-> hiv2s = 0

Summary statistics: p50, p25, p75
by categories of: outcome (Outcome)

outcome	new1band	new2band
Alive	1.55	.41
	.34	.13
	4.28	1.43
Died	2.165	1.025
	.24	.27
	4.985	3.3
Total	1.6	.49
	.28	.17
	4.41	1.98

-> hiv2s = 1

Summary statistics: p50, p25, p75
by categories of: outcome (Outcome)

outcome	new1band	new2band
Alive	1.56	.19
	.41	.035
	4.73	.99
Died	1.65	1.245
	.25	.35
	4.96	3.5
Total	1.58	.49
	.34	.07
	4.73	1.47

```
6 . bysort hiv2: tabstat  band1per band2perc if hiv2s!=., by(outcome) stats (p50 p25 p75)
```

-> hiv2s = 0

Summary statistics: p50, p25, p75
by categories of: outcome (Outcome)

outcome	band1p~c	band2p~c
Alive	13.04348	5.431573
	4.935065	.9756098
	26.89655	12
Died	14.04442	10.5
	1.501454	6.010929
	29.97014	25.95838
Total	13.96825	6.964619
	3.04878	.980768
	26.98113	15.98527

-> hiv2s = 1

Summary statistics: p50, p25, p75
by categories of: outcome (Outcome)

outcome	band1p~c	band2p~c
Alive	19.90291	2.025195
	6.8	.472973
	30.10101	11
Died	17.02335	16.9746

```
         |         8         10
         |  27.98527   25.95745
---------+----------------------
   Total |  19.00436         10
         |  7.995619   .9876543
         |  28.04124   16.98842
--------------------------------

  7 . bysort hiv2: tabstat new1plat new2plat if hiv2s!=., by(outcome) stats (p50 p25 p75)

---------------------------------------------------------------------------------------------
-> hiv2s = 0

Summary statistics: p50, p25, p75
  by categories of: outcome (Outcome)

 outcome |  new1plat  new2plat
---------+--------------------
   Alive |     194.5     229.5
         |        66       100
         |       394       472
---------+--------------------
    Died |        60        37
         |        21        11
         |       136       142
---------+--------------------
   Total |       140       181
         |        44        56
         |       308       430
------------------------------

---------------------------------------------------------------------------------------------
-> hiv2s = 1

Summary statistics: p50, p25, p75
  by categories of: outcome (Outcome)

 outcome |  new1plat  new2plat
---------+--------------------
   Alive |        94     132.5
         |        22        45
         |       241     233.5
---------+--------------------
    Died |        42      63.5
         |        11        17
         |       140       143
---------+--------------------
   Total |      60.5      98.5
         |        18        36
         |     182.5       204
------------------------------

  8 .
  9 . *Hypothesis testing

 10 .
 11 . *HIV negative - alive

 12 . signrank new1wcc=new2wcc if hiv2s==0 & outcome==0

Wilcoxon signed-rank test

        sign |      obs   sum ranks    expected
-------------+---------------------------------
    positive |       55      2699.5      2575.5
    negative |       46      2451.5      2575.5
        zero |        0           0           0
-------------+---------------------------------
         all |      101        5151        5151

unadjusted variance    87137.75
adjustment for ties       -0.38
adjustment for zeros       0.00
                       --------
adjusted variance      87137.38

Ho: new1wcc = new2wcc
             z =   0.420
    Prob > |z| =   0.6744
    Exact Prob =   0.6763

 13 . signrank new1neut=new2neut if hiv2s==0 & outcome==0

Wilcoxon signed-rank test

        sign |      obs   sum ranks    expected
```

```
              |
--------------+---------------------------------
     positive |        31       989.5         855
     negative |        26       720.5         855
         zero |         1           1           1
--------------+---------------------------------
          all |        58        1711        1711

unadjusted variance    16682.25
adjustment for ties       -0.12
adjustment for zeros      -0.25
                     ----------
adjusted variance      16681.88

Ho: new1neut = new2neut
             z =   1.041
    Prob > |z| =   0.2977
    Exact Prob =   0.3010
```

```
14 . signrank new1band=new2band if hiv2s==0 & outcome==0

Wilcoxon signed-rank test

         sign |      obs   sum ranks    expected
--------------+---------------------------------
     positive |       37        1262       850.5
     negative |       17         439       850.5
         zero |        4          10          10
--------------+---------------------------------
          all |       58        1711        1711

unadjusted variance    16682.25
adjustment for ties        0.00
adjustment for zeros      -7.50
                     ----------
adjusted variance      16674.75

Ho: new1band = new2band
             z =   3.187
    Prob > |z| =   0.0014
    Exact Prob =   0.0012
```

```
15 . signrank band1perc=band2perc if hivs==0 & outcome==0

Wilcoxon signed-rank test

         sign |      obs   sum ranks    expected
--------------+---------------------------------
     positive |       37        1064       687.5
     negative |       13         311       687.5
         zero |        2           3           3
--------------+---------------------------------
          all |       52        1378        1378

unadjusted variance    12057.50
adjustment for ties        0.00
adjustment for zeros      -1.25
                     ----------
adjusted variance      12056.25

Ho: band1perc = band2perc
             z =   3.429
    Prob > |z| =   0.0006
    Exact Prob =   0.0004
```

```
16 . signrank new1plat=new2plat if hiv2s==0 & outcome==0

Wilcoxon signed-rank test

         sign |      obs   sum ranks    expected
--------------+---------------------------------
     positive |       33        1295        2328
     negative |       63        3361        2328
         zero |        0           0           0
--------------+---------------------------------
          all |       96        4656        4656

unadjusted variance    74884.00
adjustment for ties       -2.62
adjustment for zeros       0.00
                     ----------
adjusted variance      74881.38

Ho: new1plat = new2plat
             z =  -3.775
    Prob > |z| =   0.0002
    Exact Prob =   0.0001
```

```
17 .
18 . *HIV negative - dead

19 . signrank new1wcc=new2wcc if hiv2s==0 & outcome==1

   Wilcoxon signed-rank test

           sign |      obs   sum ranks    expected
   -------------+---------------------------------
       positive |       15         247       232.5
       negative |       15         218       232.5
           zero |        0           0           0
   -------------+---------------------------------
            all |       30         465         465

   unadjusted variance     2363.75
   adjustment for ties        0.00
   adjustment for zeros       0.00
                          ----------
   adjusted variance       2363.75

   Ho: new1wcc = new2wcc
                z =   0.298
       Prob > |z| =   0.7655
       Exact Prob =   0.7766

20 . signrank new1neut=new2neut if hiv2s==0 & outcome==1

   Wilcoxon signed-rank test

           sign |      obs   sum ranks    expected
   -------------+---------------------------------
       positive |        8         108         126
       negative |       13         144         126
           zero |        1           1           1
   -------------+---------------------------------
            all |       22         253         253

   unadjusted variance      948.75
   adjustment for ties        0.00
   adjustment for zeros      -0.25
                          ----------
   adjusted variance        948.50

   Ho: new1neut = new2neut
                z =  -0.584
       Prob > |z| =   0.5589
       Exact Prob =   0.5769

21 . signrank new1band=new2band if hiv2s==0 & outcome==1

   Wilcoxon signed-rank test

           sign |      obs   sum ranks    expected
   -------------+---------------------------------
       positive |       11         148         125
       negative |        9         102         125
           zero |        2           3           3
   -------------+---------------------------------
            all |       22         253         253

   unadjusted variance      948.75
   adjustment for ties        0.00
   adjustment for zeros      -1.25
                          ----------
   adjusted variance        947.50

   Ho: new1band = new2band
                z =   0.747
       Prob > |z| =   0.4549
       Exact Prob =   0.4724

22 . signrank band1perc=band2perc if hivs==0 & outcome==1

   Wilcoxon signed-rank test

           sign |      obs   sum ranks    expected
   -------------+---------------------------------
       positive |       11         121          75
       negative |        4          29          75
           zero |        2           3           3
   -------------+---------------------------------
            all |       17         153         153

   unadjusted variance      446.25
```

```
   adjustment for ties        0.00
   adjustment for zeros      -1.25
                         ----------
   adjusted variance        445.00

   Ho: band1perc = band2perc
                z =   2.181
       Prob > |z| =   0.0292
       Exact Prob =   0.0283

23 . signrank new1plat=new2plat if hiv2s==0 & outcome==1

   Wilcoxon signed-rank test

           sign |      obs   sum ranks    expected
   -------------+---------------------------------
       positive |       20         299       217.5
       negative |        9         136       217.5
           zero |        0           0           0
   -------------+---------------------------------
            all |       29         435         435

   unadjusted variance     2138.75
   adjustment for ties        0.00
   adjustment for zeros       0.00
                         ----------
   adjusted variance       2138.75

   Ho: new1plat = new2plat
                z =   1.762
       Prob > |z| =   0.0780
       Exact Prob =   0.0798

24 .
25 .
26 . *HIV positive- alive

27 . signrank new1wcc=new2wcc if hiv2s==1 & outcome==0

   Wilcoxon signed-rank test

           sign |      obs   sum ranks    expected
   -------------+---------------------------------
       positive |       13         191         203
       negative |       15         215         203
           zero |        0           0           0
   -------------+---------------------------------
            all |       28         406         406

   unadjusted variance     1928.50
   adjustment for ties        0.00
   adjustment for zeros       0.00
                         ----------
   adjusted variance       1928.50

   Ho: new1wcc = new2wcc
                z =  -0.273
       Prob > |z| =   0.7847
       Exact Prob =   0.7966

28 . signrank new1neut=new2neut if hiv2s==1 & outcome==0

   Wilcoxon signed-rank test

           sign |      obs   sum ranks    expected
   -------------+---------------------------------
       positive |        2          14        38.5
       negative |        9          63        38.5
           zero |        1           1           1
   -------------+---------------------------------
            all |       12          78          78

   unadjusted variance      162.50
   adjustment for ties        0.00
   adjustment for zeros      -0.25
                         ----------
   adjusted variance        162.25

   Ho: new1neut = new2neut
                z =  -1.923
       Prob > |z| =   0.0544
       Exact Prob =   0.0576

29 . signrank new1band=new2band if hiv2s==1 & outcome==0

   Wilcoxon signed-rank test
```

```
       sign |      obs   sum ranks    expected
-------------+---------------------------------
    positive |        8          63        38.5
    negative |        3          14        38.5
        zero |        1           1           1
-------------+---------------------------------
         all |       12          78          78

unadjusted variance      162.50
adjustment for ties        0.00
adjustment for zeros      -0.25
                     ----------
adjusted variance        162.25

Ho: new1band = new2band
             z =   1.923
    Prob > |z| =   0.0544
    Exact Prob =   0.0576
```

```
30 . signrank band1perc=band2perc if hivs==1 & outcome==0

   Wilcoxon signed-rank test

       sign |      obs   sum ranks    expected
-------------+---------------------------------
    positive |        4          14           7
    negative |        0           0           7
        zero |        1           1           1
-------------+---------------------------------
         all |        5          15          15

unadjusted variance       13.75
adjustment for ties        0.00
adjustment for zeros      -0.25
                     ----------
adjusted variance         13.50

Ho: band1perc = band2perc
             z =   1.905
    Prob > |z| =   0.0568
    Exact Prob =   0.1250
```

```
31 . signrank new1plat=new2plat if hiv2s==1 & outcome==0

   Wilcoxon signed-rank test

       sign |      obs   sum ranks    expected
-------------+---------------------------------
    positive |       10         111         203
    negative |       18         295         203
        zero |        0           0           0
-------------+---------------------------------
         all |       28         406         406

unadjusted variance     1928.50
adjustment for ties       -0.25
adjustment for zeros       0.00
                     ----------
adjusted variance       1928.25

Ho: new1plat = new2plat
             z =  -2.095
    Prob > |z| =   0.0362
    Exact Prob =   0.0352
```

```
32 .
33 . *HIV positive - dead

34 . signrank new1wcc=new2wcc if hiv2s==1 & outcome==1

   Wilcoxon signed-rank test

       sign |      obs   sum ranks    expected
-------------+---------------------------------
    positive |       11          86        85.5
    negative |        7          85        85.5
        zero |        0           0           0
-------------+---------------------------------
         all |       18         171         171

unadjusted variance      527.25
adjustment for ties        0.00
adjustment for zeros       0.00
                     ----------
adjusted variance        527.25
```

```
Ho: new1wcc = new2wcc
             z =   0.022
    Prob > |z| =   0.9826
    Exact Prob =   1.0000

35 . signrank new1neut=new2neut if hiv2s==1 & outcome==1

Wilcoxon signed-rank test

        sign │      obs   sum ranks    expected
─────────────┼─────────────────────────────────
    positive │        9          66        52.5
    negative │        5          39        52.5
        zero │        0           0           0
─────────────┼─────────────────────────────────
         all │       14         105         105

unadjusted variance      253.75
adjustment for ties        0.00
adjustment for zeros       0.00
                     ──────────
adjusted variance        253.75

Ho: new1neut = new2neut
             z =   0.847
    Prob > |z| =   0.3967
    Exact Prob =   0.4263

36 . signrank new1band=new2band if hiv2s==1 & outcome==1

Wilcoxon signed-rank test

        sign │      obs   sum ranks    expected
─────────────┼─────────────────────────────────
    positive │        7          54          52
    negative │        6          50          52
        zero │        1           1           1
─────────────┼─────────────────────────────────
         all │       14         105         105

unadjusted variance      253.75
adjustment for ties        0.00
adjustment for zeros      -0.25
                     ──────────
adjusted variance        253.50

Ho: new1band = new2band
             z =   0.126
    Prob > |z| =   0.9000
    Exact Prob =   0.9272

37 . signrank band1perc=band2perc if hivs==1 & outcome==1

Wilcoxon signed-rank test

        sign │      obs   sum ranks    expected
─────────────┼─────────────────────────────────
    positive │        3           8         7.5
    negative │        2           7         7.5
        zero │        0           0           0
─────────────┼─────────────────────────────────
         all │        5          15          15

unadjusted variance       13.75
adjustment for ties        0.00
adjustment for zeros       0.00
                     ──────────
adjusted variance         13.75

Ho: band1perc = band2perc
             z =   0.135
    Prob > |z| =   0.8927
    Exact Prob =   1.0000

38 . signrank new1plat=new2plat if hiv2s==1 & outcome==1

Wilcoxon signed-rank test

        sign │      obs   sum ranks    expected
─────────────┼─────────────────────────────────
    positive │        8          73        76.5
    negative │        9          80        76.5
        zero │        0           0           0
─────────────┼─────────────────────────────────
         all │       17         153         153
```

```
    unadjusted variance      446.25
    adjustment for ties       -0.12
    adjustment for zeros       0.00
                           ----------
    adjusted variance        446.12

    Ho: new1plat = new2plat
                 z =  -0.166
        Prob > |z| =   0.8684
        Exact Prob =   0.8803

 39 .
 40 .
```
