# Supplementary material for: Association of full blood count findings with risk of mortality in children with Klebsiella pneumoniae bloodstream infection at a south african children’s hospital
Source: BMC Pediatr. 2023 Jun 17;23:302. doi: 10.1186/s12887-023-04104-z (PMC10276429; doi:10.1186/s12887-023-04104-z)
Supplement: Supplementary file 2 — Supplementary Material 2 [file 12887_2023_4104_MOESM2_ESM.pdf]

```
1 .
2 . * Descriptions

3 . bysort hiv2: tabstat new1wcc new2wcc if hiv2s!=., by(outcome) stats (p50 p25 p75)

------------------------------------------------------------------------------------
-> hiv2s = 0

Summary statistics: p50, p25, p75
  by categories of: outcome (Outcome)

 outcome |  new1wcc   new2wcc
---------+--------------------
   Alive |     11.7     11.13
         |      7.2      8.19
         |    20.07     16.89
---------+--------------------
    Died |    12.57     13.08
         |      5.9       5.2
         |    21.87      23.6
---------+--------------------
   Total |     11.9    11.545
         |      6.6      7.68
         |       21     17.72
------------------------------

------------------------------------------------------------------------------------
-> hiv2s = 1

Summary statistics: p50, p25, p75
  by categories of: outcome (Outcome)

 outcome |  new1wcc   new2wcc
---------+--------------------
   Alive |    9.305       9.9
         |     4.95         7
         |     13.2     12.55
---------+--------------------
    Died |     8.82      9.35
         |        3       4.1
         |    20.26     12.08
---------+--------------------
   Total |     8.82       9.9
         |        4       5.7
         |    16.57      12.4
------------------------------

4 . bysort hiv2: tabstat new1neut new2neut if hiv2s!=., by(outcome) stats (p50 p25 p75)

------------------------------------------------------------------------------------
-> hiv2s = 0

Summary statistics: p50, p25, p75
  by categories of: outcome (Outcome)

 outcome | new1neut  new2neut
---------+--------------------
   Alive |     5.61      4.42
         |      2.8       2.1
         |   10.275      8.33
---------+--------------------
    Died |     4.63     6.325
         |      .76      1.33
         |    12.69      15.1
---------+--------------------
   Total |     5.09      4.42
         |      2.2      1.84
         |    10.37     10.06
------------------------------

------------------------------------------------------------------------------------
-> hiv2s = 1

Summary statistics: p50, p25, p75
  by categories of: outcome (Outcome)

 outcome | new1neut  new2neut
---------+--------------------
   Alive |     3.63     3.995
         |      .99      2.31
         |     7.28      5.56
---------+--------------------
    Died |      2.9      3.41
         |      .62      1.41
         |     8.45      6.29
```

```
    Total |      3.36     3.735
          |      .865      1.72
          |     7.865      6.02
---------------------------------

5 . bysort hiv2: tabstat new1band new2band if hiv2s!=., by(outcome) stats (p50 p25 p75)

-----------------------------------------------------------------------------------------------------------
-> hiv2s = 0

Summary statistics: p50, p25, p75
  by categories of: outcome (Outcome)

  outcome |  new1band  new2band
----------+--------------------
    Alive |     1.385       .41
          |      .305       .13
          |     3.645      1.43
----------+--------------------
     Died |      1.81      .495
          |       .22       .18
          |      4.37      2.31
----------+--------------------
    Total |       1.4       .44
          |       .27       .13
          |      3.66      1.53
-------------------------------

-----------------------------------------------------------------------------------------------------------
-> hiv2s = 1

Summary statistics: p50, p25, p75
  by categories of: outcome (Outcome)

  outcome |  new1band  new2band
----------+--------------------
    Alive |     1.525       .19
          |       .41      .035
          |      4.18       .99
----------+--------------------
     Died |      1.65     1.245
          |       .25       .35
          |      4.96       3.5
----------+--------------------
    Total |      1.57       .49
          |       .34       .07
          |       4.6      1.47
-------------------------------

6 . bysort hiv2: tabstat  band1per band2perc if hiv2s!=., by(outcome) stats (p50 p25 p75)

-----------------------------------------------------------------------------------------------------------
-> hiv2s = 0

Summary statistics: p50, p25, p75
  by categories of: outcome (Outcome)

  outcome |  band1p~c  band2p~c
----------+--------------------
    Alive |  13.47349  5.431573
          |  3.011608  .9760301
          |  26.45713  12.50106
----------+--------------------
     Died |   13.9967  7.982301
          |  2.012809  .9788769
          |  27.01835  13.98305
----------+--------------------
    Total |  13.96825  5.990539
          |  3.010033  .9764503
          |  26.89655  13.00211
-------------------------------

-----------------------------------------------------------------------------------------------------------
-> hiv2s = 1

Summary statistics: p50, p25, p75
  by categories of: outcome (Outcome)

  outcome |  band1p~c  band2p~c
----------+--------------------
    Alive |  19.45364  2.025195
          |       6.8   .472973
          |  29.01099        11
----------+--------------------
     Died |  17.02335   16.9746
```

```
         |         8        10
         |  27.98527  25.95745
---------+--------------------
   Total |  18.50218        10
         |  7.970823  .9876543
         |  28.01325  16.98842
------------------------------

 7 . bysort hiv2: tabstat new1plat new2plat if hiv2s!=., by(outcome) stats (p50 p25 p75)

------------------------------------------------------------------------------------------------------------------------------------------------------
-> hiv2s = 0

Summary statistics: p50, p25, p75
  by categories of: outcome (Outcome)

 outcome |  new1plat  new2plat
---------+--------------------
   Alive |       204       239
         |        78       110
         |     383.5       499
---------+--------------------
    Died |        61      40.5
         |        18        16
         |       136       142
---------+--------------------
   Total |     142.5       209
         |        48        64
         |       313       455
------------------------------

------------------------------------------------------------------------------------------------------------------------------------------------------
-> hiv2s = 1

Summary statistics: p50, p25, p75
  by categories of: outcome (Outcome)

 outcome |  new1plat  new2plat
---------+--------------------
   Alive |      92.5     132.5
         |        22        45
         |       238     233.5
---------+--------------------
    Died |        42      63.5
         |        11        17
         |       140       143
---------+--------------------
   Total |        59      98.5
         |        18        36
         |       173       204
------------------------------

 8 .
 9 . *Hypothesis testing

10 .
11 . *HIV negative - alive

12 . signrank new1wcc=new2wcc if hiv2s==0 & outcome==0

Wilcoxon signed-rank test

        sign |      obs   sum ranks    expected
-------------+---------------------------------
    positive |       48      2053.5        1958
    negative |       40      1862.5        1958
        zero |        0           0           0
-------------+---------------------------------
         all |       88        3916        3916

unadjusted variance    57761.00
adjustment for ties       -0.25
adjustment for zeros       0.00
                     ----------
adjusted variance      57760.75

Ho: new1wcc = new2wcc
             z =   0.397
    Prob > |z| =   0.6911
    Exact Prob =   0.6933

13 . signrank new1neut=new2neut if hiv2s==0 & outcome==0

Wilcoxon signed-rank test

        sign |      obs   sum ranks    expected
```

positive	29	829.5	688.5
negative	22	547.5	688.5
zero	1	1	1
all	52	1378	1378

```
unadjusted variance    12057.50
adjustment for ties       -0.12
adjustment for zeros      -0.25
                     ----------
adjusted variance      12057.12

Ho: new1neut = new2neut
             z =   1.284
    Prob > |z| =   0.1991
    Exact Prob =   0.2017
```

14 . signrank new1band=new2band if hiv2s==0 & outcome==0

Wilcoxon signed-rank test

sign	obs	sum ranks	expected
positive	33	1026	684
negative	15	342	684
zero	4	10	10
all	52	1378	1378

```
unadjusted variance    12057.50
adjustment for ties        0.00
adjustment for zeros      -7.50
                     ----------
adjusted variance      12050.00

Ho: new1band = new2band
             z =   3.116
    Prob > |z| =   0.0018
    Exact Prob =   0.0015
```

15 . signrank band1perc=band2perc if hivs==0 & outcome==0

Wilcoxon signed-rank test

sign	obs	sum ranks	expected
positive	32	826	539
negative	12	252	539
zero	2	3	3
all	46	1081	1081

```
unadjusted variance     8377.75
adjustment for ties        0.00
adjustment for zeros      -1.25
                     ----------
adjusted variance       8376.50

Ho: band1perc = band2perc
             z =   3.136
    Prob > |z| =   0.0017
    Exact Prob =   0.0013
```

16 . signrank new1plat=new2plat if hiv2s==0 & outcome==0

Wilcoxon signed-rank test

sign	obs	sum ranks	expected
positive	28	919.5	1743
negative	55	2566.5	1743
zero	0	0	0
all	83	3486	3486

```
unadjusted variance    48513.50
adjustment for ties       -2.12
adjustment for zeros       0.00
                     ----------
adjusted variance      48511.38

Ho: new1plat = new2plat
             z =  -3.739
    Prob > |z| =   0.0002
    Exact Prob =   0.0001
```

```
17 .
18 . *HIV negative - dead

19 . signrank new1wcc=new2wcc if hiv2s==0 & outcome==1

   Wilcoxon signed-rank test

           sign |      obs   sum ranks    expected
   -------------+---------------------------------
       positive |       12         152       126.5
       negative |       10         101       126.5
           zero |        0           0           0
   -------------+---------------------------------
            all |       22         253         253

   unadjusted variance      948.75
   adjustment for ties        0.00
   adjustment for zeros       0.00
                          ----------
   adjusted variance        948.75

   Ho: new1wcc = new2wcc
                z =   0.828
       Prob > |z| =   0.4077
       Exact Prob =   0.4245

20 . signrank new1neut=new2neut if hiv2s==0 & outcome==1

   Wilcoxon signed-rank test

           sign |      obs   sum ranks    expected
   -------------+---------------------------------
       positive |        7          65        67.5
       negative |        8          70        67.5
           zero |        1           1           1
   -------------+---------------------------------
            all |       16         136         136

   unadjusted variance      374.00
   adjustment for ties        0.00
   adjustment for zeros      -0.25
                          ----------
   adjusted variance        373.75

   Ho: new1neut = new2neut
                z =  -0.129
       Prob > |z| =   0.8971
       Exact Prob =   0.9199

21 . signrank new1band=new2band if hiv2s==0 & outcome==1

   Wilcoxon signed-rank test

           sign |      obs   sum ranks    expected
   -------------+---------------------------------
       positive |       10         102        66.5
       negative |        4          31        66.5
           zero |        2           3           3
   -------------+---------------------------------
            all |       16         136         136

   unadjusted variance      374.00
   adjustment for ties        0.00
   adjustment for zeros      -1.25
                          ----------
   adjusted variance        372.75

   Ho: new1band = new2band
                z =   1.839
       Prob > |z| =   0.0660
       Exact Prob =   0.0691

22 . signrank band1perc=band2perc if hivs==0 & outcome==1

   Wilcoxon signed-rank test

           sign |      obs   sum ranks    expected
   -------------+---------------------------------
       positive |        9          86          51
       negative |        3          16          51
           zero |        2           3           3
   -------------+---------------------------------
            all |       14         105         105

   unadjusted variance      253.75
```

```
   adjustment for ties        0.00
   adjustment for zeros      -1.25
                          ----------
   adjusted variance        252.50

   Ho: band1perc = band2perc
                z =   2.203
       Prob > |z| =   0.0276
       Exact Prob =   0.0264

23 . signrank new1plat=new2plat if hiv2s==0 & outcome==1

   Wilcoxon signed-rank test

           sign |      obs   sum ranks    expected
   -------------+---------------------------------
       positive |       15         171       115.5
       negative |        6          60       115.5
           zero |        0           0           0
   -------------+---------------------------------
            all |       21         231         231

   unadjusted variance      827.75
   adjustment for ties        0.00
   adjustment for zeros       0.00
                          ----------
   adjusted variance        827.75

   Ho: new1plat = new2plat
                z =   1.929
       Prob > |z| =   0.0537
       Exact Prob =   0.0547

24 .
25 .
26 . *HIV positive- alive

27 . signrank new1wcc=new2wcc if hiv2s==1 & outcome==0

   Wilcoxon signed-rank test

           sign |      obs   sum ranks    expected
   -------------+---------------------------------
       positive |       13         191         203
       negative |       15         215         203
           zero |        0           0           0
   -------------+---------------------------------
            all |       28         406         406

   unadjusted variance     1928.50
   adjustment for ties        0.00
   adjustment for zeros       0.00
                          ----------
   adjusted variance       1928.50

   Ho: new1wcc = new2wcc
                z =  -0.273
       Prob > |z| =   0.7847
       Exact Prob =   0.7966

28 . signrank new1neut=new2neut if hiv2s==1 & outcome==0

   Wilcoxon signed-rank test

           sign |      obs   sum ranks    expected
   -------------+---------------------------------
       positive |        2          14        38.5
       negative |        9          63        38.5
           zero |        1           1           1
   -------------+---------------------------------
            all |       12          78          78

   unadjusted variance      162.50
   adjustment for ties        0.00
   adjustment for zeros      -0.25
                          ----------
   adjusted variance        162.25

   Ho: new1neut = new2neut
                z =  -1.923
       Prob > |z| =   0.0544
       Exact Prob =   0.0576

29 . signrank new1band=new2band if hiv2s==1 & outcome==0

   Wilcoxon signed-rank test
```

```
        sign │      obs   sum ranks    expected
─────────────┼─────────────────────────────────
    positive │        8          63        38.5
    negative │        3          14        38.5
        zero │        1           1           1
─────────────┼─────────────────────────────────
         all │       12          78          78

unadjusted variance      162.50
adjustment for ties        0.00
adjustment for zeros      -0.25
                     ──────────
adjusted variance        162.25

Ho: new1band = new2band
             z =   1.923
    Prob > |z| =   0.0544
    Exact Prob =   0.0576

30 . signrank band1perc=band2perc if hivs==1 & outcome==0

Wilcoxon signed-rank test

        sign │      obs   sum ranks    expected
─────────────┼─────────────────────────────────
    positive │        4          14           7
    negative │        0           0           7
        zero │        1           1           1
─────────────┼─────────────────────────────────
         all │        5          15          15

unadjusted variance       13.75
adjustment for ties        0.00
adjustment for zeros      -0.25
                     ──────────
adjusted variance         13.50

Ho: band1perc = band2perc
             z =   1.905
    Prob > |z| =   0.0568
    Exact Prob =   0.1250

31 . signrank new1plat=new2plat if hiv2s==1 & outcome==0

Wilcoxon signed-rank test

        sign │      obs   sum ranks    expected
─────────────┼─────────────────────────────────
    positive │       10         111         203
    negative │       18         295         203
        zero │        0           0           0
─────────────┼─────────────────────────────────
         all │       28         406         406

unadjusted variance     1928.50
adjustment for ties       -0.25
adjustment for zeros       0.00
                     ──────────
adjusted variance       1928.25

Ho: new1plat = new2plat
             z =  -2.095
    Prob > |z| =   0.0362
    Exact Prob =   0.0352

32 .
33 . *HIV positive - dead

34 . signrank new1wcc=new2wcc if hiv2s==1 & outcome==1

Wilcoxon signed-rank test

        sign │      obs   sum ranks    expected
─────────────┼─────────────────────────────────
    positive │       11          86        85.5
    negative │        7          85        85.5
        zero │        0           0           0
─────────────┼─────────────────────────────────
         all │       18         171         171

unadjusted variance      527.25
adjustment for ties        0.00
adjustment for zeros       0.00
                     ──────────
adjusted variance        527.25
```

```
Ho: new1wcc = new2wcc
             z =   0.022
    Prob > |z| =   0.9826
    Exact Prob =   1.0000

35 . signrank new1neut=new2neut if hiv2s==1 & outcome==1

   Wilcoxon signed-rank test

           sign |      obs   sum ranks    expected
   -------------+---------------------------------
       positive |        9          66        52.5
       negative |        5          39        52.5
           zero |        0           0           0
   -------------+---------------------------------
            all |       14         105         105

   unadjusted variance      253.75
   adjustment for ties        0.00
   adjustment for zeros       0.00
                          --------
   adjusted variance        253.75

   Ho: new1neut = new2neut
             z =   0.847
    Prob > |z| =   0.3967
    Exact Prob =   0.4263

36 . signrank new1band=new2band if hiv2s==1 & outcome==1

   Wilcoxon signed-rank test

           sign |      obs   sum ranks    expected
   -------------+---------------------------------
       positive |        7          54          52
       negative |        6          50          52
           zero |        1           1           1
   -------------+---------------------------------
            all |       14         105         105

   unadjusted variance      253.75
   adjustment for ties        0.00
   adjustment for zeros      -0.25
                          --------
   adjusted variance        253.50

   Ho: new1band = new2band
             z =   0.126
    Prob > |z| =   0.9000
    Exact Prob =   0.9272

37 . signrank band1perc=band2perc if hivs==1 & outcome==1

   Wilcoxon signed-rank test

           sign |      obs   sum ranks    expected
   -------------+---------------------------------
       positive |        2           3         1.5
       negative |        0           0         1.5
           zero |        0           0           0
   -------------+---------------------------------
            all |        2           3           3

   unadjusted variance        1.25
   adjustment for ties        0.00
   adjustment for zeros       0.00
                          --------
   adjusted variance          1.25

   Ho: band1perc = band2perc
             z =   1.342
    Prob > |z| =   0.1797
    Exact Prob =   0.5000

38 . signrank new1plat=new2plat if hiv2s==1 & outcome==1

   Wilcoxon signed-rank test

           sign |      obs   sum ranks    expected
   -------------+---------------------------------
       positive |        8          73        76.5
       negative |        9          80        76.5
           zero |        0           0           0
   -------------+---------------------------------
            all |       17         153         153
```

```
   unadjusted variance      446.25
   adjustment for ties       -0.12
   adjustment for zeros       0.00
                          ----------
   adjusted variance        446.12

   Ho: new1plat = new2plat
                z =  -0.166
       Prob > |z| =   0.8684
       Exact Prob =   0.8803

39 .
40 .
```
